# Transcriptomic Analysis of *Calonectria pseudoreteaudii* during Various Stages of *Eucalyptus* Infection

**DOI:** 10.1371/journal.pone.0169598

**Published:** 2017-01-10

**Authors:** Xiaozhen Ye, Hongyi Liu, Yajie Jin, Mengmeng Guo, Aizhen Huang, Quanzhu Chen, Wenshuo Guo, Feiping Zhang, Lizhen Feng

**Affiliations:** 1 Forestry College, Fujian Agriculture and Forestry University, Fuzhou, China; 2 Jinshan College, Fujian Agriculture and Forestry University, Fuzhou, China; 3 Institute of Forestry Protection, Fujian Agriculture and Forestry University, Fuzhou, China; National Renewable Energy Laboratory, UNITED STATES

## Abstract

*Eucalyptus* leaf blight caused by *Calonectria* spp. is a serious disease in *Eucalyptus* seedling and plantations. However, the molecular mechanisms of the infection process and pathogenesis of *Calonectria* to *Eucalyptus* is not well-studied. In this study, we analyzed the transcriptomes of *C*. *pseudoreteaudii* at three stages of *Eucalyptus* leaf infection, and in mycelium grown in potato dextrose broth using Illumina RNA-Seq technology. We identified 161 differentially expressed genes between *C*. *pseudoreteaudii* from leaf and mycelium grown in potato dextrose broth. GO and KEGG enrichment analyses of these genes suggested that they were mainly involved in oxidoreductase activity, hydrolase activity, and transmembrane transporter activity. Most of the differentially expressed genes at the early infection stage were upregulated. These upregulated genes were mainly involved in cell wall hydrolysis and toxin synthesis, suggesting a role for toxin and cell wall hydrolases in the establishment of *Calonectria* leaf blight. Genes related to detoxification of phytoalexins were continually upregulated during infection. The candidate effectors and putative pathogenicity determinants identified in this study will help in the functional analysis of *C*. *pseudoreteaudii* virulence and pathogenicity.

## Introduction

*Eucalyptus* trees are the most valuable hardwood trees in the world known for their fast growth and superior wood properties. *Calonectria* spp. is one of the most aggressive pathogens affecting *Eucalyptus* seedling and plantations [1,2]. It infects the leaves and branches of *Eucalyptus* causing leaf blotch, shoot blight, stem canker, and total defoliation, impeding the development of *Eucalyptus* [3]. Infection by this fungus causes annual yield losses averaging upwards of $ 7.8 million in the Fujian Province [4].

Many studies have been conducted on the identification and taxonomy of this pathogen [5,6]. The virulence, biological characteristics, and control measures of some important *Calonectria* spp. have been described in our previous studies[7–9]. This genus includes more than 68 species, most of which have strong pathogenicity on *Eucalyptus*. At least seven species of *Calonectria* have been found in *Eucalyptus* seedling nursery and plantation in the Southern provinces of China [3,8,10,11], of which *C*. *pseudoreteaudii* is the first record specie in Fujian Province [8]. It is also the most widely distributed and aggressive specie because of its excellent temperature adaptability [8,12]. However, the infection process and pathogenesis of *C*. *pseudoreteaudii* on *Eucalyptus* remain unclear.

In our previous study, we determined the resistance mechanism of different *Eucalyptus* clones against *C*. *pseudoreteaudii* based on their anatomical structure, physiology, and resistance-related genes [13–19]. Our results showed that the wax layer and the cuticle were the first physical barrier of the resistant clones against pathogen infection. Moreover, after infection with *C*. *pseudoreteaudii*, the resistant clones enhanced the defense response by increasing the synthesis of antifungal compounds, such as polyphenols and flavonoid.

Advances in RNA-seq technology enable comparative transcriptomic analysis to isolate pathogen-related genes and determine crucial metabolic pathways, which act important roles in the host-pathogen interactions. Transcriptome analysis helped identify genes involved in ferrous iron uptake or the response to oxidative stress of *Clostridium difficile* in vivo and in vitro [20]. Also, a hypothetical proteins were observed to highly and specifically upregulate in vivo, and finally identified as a putative colonization factor. The comprehensive transcriptome analysis of *Cronartium ribicola* facilitates the identification of candidate effectors and other putative pathogenicity determinants [21].

In the present study, we analyzed the transcriptomes of *C*. *pseudoreteaudii* at three stage of infection on *Eucalyptus* leaves using the ‘next generation’ sequencing technology. The mycelium of *C*. *pseudoreteaudii* grown on PDB (potato dextrose broth) was employed as a control. The genome of *C*. *pseudoreteaudii* was used as the reference sequence (Unpublished data). Our objective was to identify some candidate factors involved in the virulence or pathogenicity of *C*. *pseudoreteaudii* on *Eucalyptus*.

## Materials and Methods

### Plant material and infection procedures

*E*. *grandis* × *E*. *tereticornis* M1 is a variety from the Southeastern part of China, which is characterized by a durable resistance to *Calonectira* spp. [22]. The tissue-cultured seedlings were grown in pots and cultivated in a climate chamber with 25–28°C, 90% humidity and a 14 h/10 h (day/night) photoperiod. *C*. *pseudoreteaudii* inoculation was performed as described previously [14]. For studying the infection process, leaves inoculated with conidia suspension were sampled at 4, 6, 8, 12, and 24 hours post inoculation (hpi). For RNA-seq analysis and qRT-PCR, the leaves were sampled at 12, 24, and 48 hpi according to the observation of the infection process by scanning electron microscopy (SEM). *C*. *pseudoreteaudii* mycelia cultured in 150 mL potato dextrose broth (PDB) medium for two days were collected as control samples. All the samples were immediately lyophilized and stored at −80°C.

### Scanning electron microscopy

Conidia germination and infection process on the leaves of *Eucalyptus* was observed by SEM. Leaves infected by *C*. *pseudoreteaudii* were cut into 3 × 3 mm pieces and treated as described by Ke et al. [23]. The samples were immersed in 2.5% glutaraldehyde prepared with 0.1 mol·L^-1^ phosphate buffer (pH 7.0) over night at 4°C. The samples were then washed with sterile water three times (10 minutes each time) and dehydrated in a graded series of 50%, 70%, 83%, 95% tert-butanol (30 min at each step), and a final step of 100% for 5 min. After that, all the samples were flooded with 100% tert-butanol at 4°C until crystallization and then transferred to a vacuum drier for drying at room temperature for three hours. Finally, the dried samples were mounted on stubs with double-sided tape and sputter-coated with a gold-palladium target (JFC-1200). Samples were observed with a JSM 6380LV (JEOL) SEM.

### Genome mapping

RNA-seq data of pathogen-infected leaves at 12 and 24 hpi was generated in a previous study [13]. The 48 hpi data that was sequenced earlier (unpublished) was used to investigate pathogen gene expression in the process of infection and colonization of *Eucalyptus*. Adapter reads in which unknown bases were more than 10%, and low-quality reads (the percentage of the low-quality bases of quality value ≤ 0.05 was more than 50% in a read) were filtered. To avoid the contamination of reads from *Eucalyptus*, clean reads of each sample were firstly mapped against the sequenced genome of *E*. *grandis* (http://phytozome.net/eucalyptus.php) using Bowtie and TopHat software [24]. Then, the remaining unmapped non-host reads were mapped to the genome of *C*. *pseudoreteaudii* (Accession number MOCD00000000) with the same software.

### DEGs analysis

Gene expression levels were calculated as FPKM (Fragments Per Kilobase of exon per Million fragments mapped) using Cufflinks [25]. To identify differentially expressed genes (DEGs), we used the Bayesian-adjusted ‘t’ statistics and performed the multiple-testing corrections of Benjamini and Hochberg. Gene with Log2foldchange ≥1 with *P* < 0.05 and FDR < 0.05 was considered as differentially expressed gene. Then, all the DEGs were mapped to gene ontology terms in the database (GO, http://www.geneontology.org/) for functional annotation and to Kyoto Encyclopedia of Genes and Genomes database (KEGG, http://www.genome.jp/kegg/pathway.html) for enrichment analysis.

### Quantitative RT-PCR verification

To validate the DEGs data obtained by RNA-Seq, six DEGs relative to pathogen virulence were selected for qRT-PCR analysis. Primers were designed with Primer Premier 5.0 and synthesized by Beijing Genomics Institute (BGI, Shenzhen, China) (S1 File). About 1 μg total RNA was used for cDNA synthesis by FastQuant RT Kit (With gDNase) (Tiangen, Beijing) according to the manufacturers’ instructions. PCR amplification was conducted in a 10 μL reaction system containing 5 μL SYBR^®^ Premix Ex Taq^™^ (Tli RNaseH Plus) (TaKaRa, Japan) and performed on a QuantStudio^™^ 6 Flex Real-Time PCR System (Applied Biosystems, USA). The cycling conditions were 95°C for 30 s followed by 45 cycling of 95°C for 5 s, 60°C for 20 s. Three biological replicates were performed for each target gene. The relative expression levels of DEGs were analyzed using the 2^-ΔΔCT^ method and normalized to the reference genes *β-tubulin*.

## Results

### Conidia germination and fungal infection

**Fig 1:**
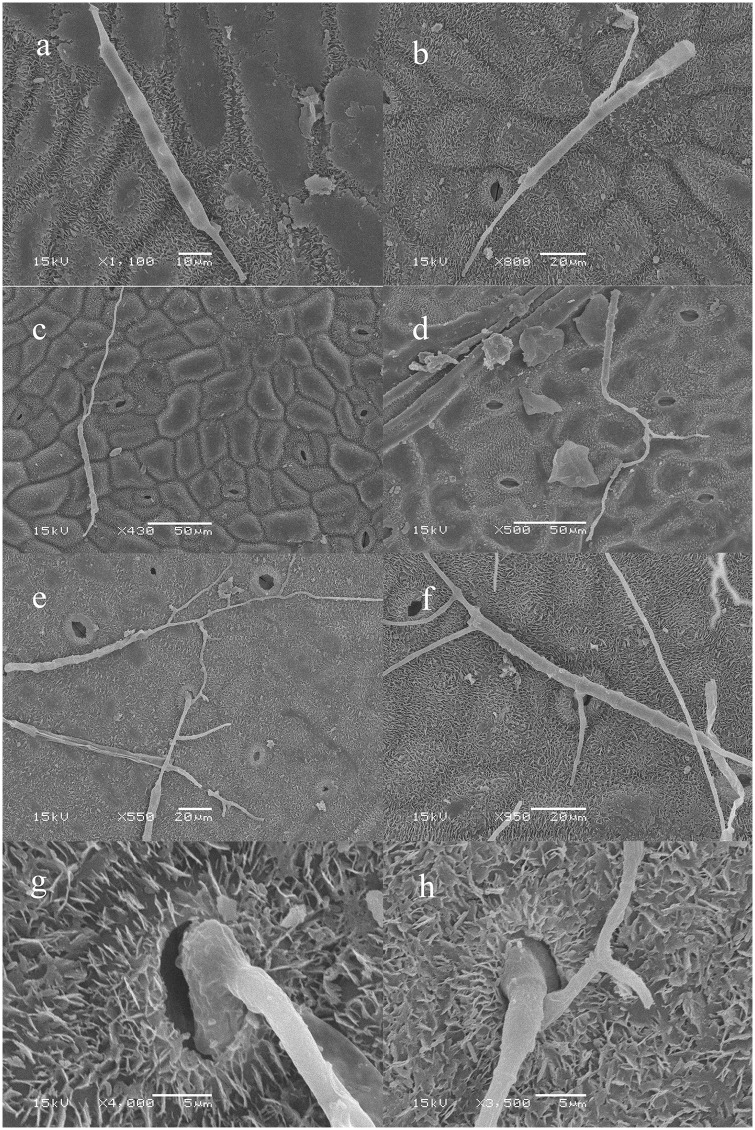
Scanning electron micrographs of the conidia germination and infection of *C*. *pseudoreteaudii* on *Eucalyptus* leaf. a-b, conidia germinated from both ends or septum at 6 hpi; c-d, each germ tube could produce multiple branches at 8 hpi; e-h, the ends of germ tubes expanded and invaded the leaves from the stoma at 12 hpi.

Scanning Electron Microscopy (SEM) showed that conidia germinated from both ends or septum at 6 hpi, and typically produced one or more germ tubes (Fig 1a and 1b). Each germ tube could produced multiple branches (Fig 1c and 1d). After 8 h of inoculation, the ends of germ tubes expanded and invaded the leaves from the stoma (Fig 1e–1h). This results corroborated a previous study by Zheng [26].

### RNA-Seq analysis

We sequenced the transcriptome of *C*. *pseudoreteaudii* in infected *Eucalyptus* at the early, middle, and later stages, and mycelium cultured in PDB for two days. Raw reads of the six samples were submitted to the Sequence Read Archive (SRA) database of NCBI (SRP090272). The 7 million single-end reads of 49 bp in length obtained from each sample are shown in Table 1. After removing reads specific to *Eucalyptus*, the remaining reads of infected tissue were mapped to the genome of *C*. *pseudoreteaudii*. We uniquely mapped 48018 (0.60%), 82612 (1.10%), 700283 (9.40%) to the genome of *C*. *pseudoreteaudii*. The number of reads mapping to the pathogen increased with the infection time. Since the number of fungal cells was low at the early stage of fungal infection, the detection of fungal transcripts was difficult.

**Table 1:** Summary of mapping results.

	Sample			
	12 hpi	24 hpi	48hpi	Control
**Total reads**	7548102	7472614	7454265	32703371
**Reads mapped to Eg**^a^ **genome**	6617772	6447543	5903236	552
**Map to Eg percentage (%)**	87.70%	86.30%	79.20%	0%
**Reads mapped to Cp**^b^ **genome**	48018	82612	700283	28792563
**Map to Cp percentage (%)**	0.60%	1.10%	9.40%	88.00%

^a^ represents *Eucalyptus grandis*,

^b^ represents *Calonectria pseudoreteaudii*

**Fig 2:**
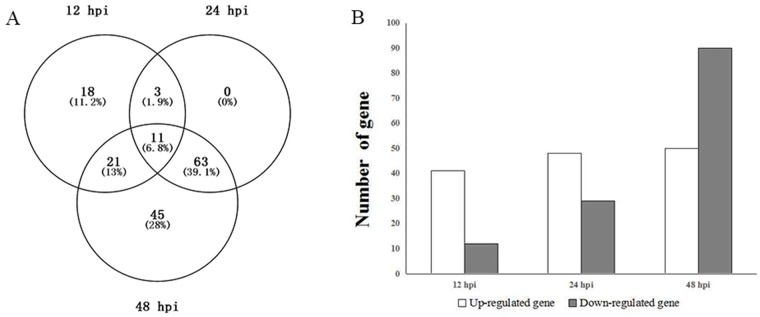
Differentially expressed genes (DEG) of *C*. *pseudoreteaudii*. A, venn diagram showed common DEGs at three infection stages. B, number of DEGs at different colonization stages of *C*. *pseudoreteaudii*.

With the parameter log2foldchange ≥1 and *P* < 0.05, 161 DEGs were detected at the three stages of *Eucalyptus* infection (Fig 2A). Among them, 11 were continuously observed at all infection stages, and they were all upregulated. There were 18, 0, and 45 DEGs at the early, middle, and later stages, respectively. All of the genes expressed at the early stage were upregulated. Several of these upregulated genes were involved in pathogen invasion. For example, a cutinase gene (Cp_Cap05169) was highly expressed at the early stage. However, the function of most other genes is unknown. There was no significant difference in the number of upregulated genes at the three stages; however, the number of down-regulated genes was higher at the later stage (Fig 2B). This indicated that *C*. *pseudoreteaudii* suffered increasing stress from the host over time.

There were 13 function known genes encoding salicylate hydroxylase, MFS transporter, phosphatase, oxidoreductase, subtilase, endopolygalacturonase, among the top 20 upregulated genes of *C*. *pseudoreteaudii* (Table 2). Most of them were reported to be involved in the infection process of pathogen like the salicylate hydroxylase gene, which was the most significantly upregulated gene. Salicylate hydroxylase can be produced by microorganisms for the degradation of salicylic acid (SA) and suppresses SA-mediated defense of plant [27]. Moreover, we annotated five genes as oxidoreductases that were associated with reactive oxygen species (ROS) scavenging.

**Table 2:** List of the 20 most up-regulated genes of *C*. *pseudoreteaudii* during infection.

Gene id	log2 (fold change)			Description
	12 hpi	24 hpi	48 hpi	
Cp_Cap03373	-	9.611	13.399	salicylate hydroxylase
Cp_Cap14235	13.005	9.018	-	-
Cp_Cap01164	-	9.600	12.458	MFS transporter
Cp_Cap07325	-	-	12.280	oxidoreductase
Cp_Cap09735	11.781	-	3.566	-
Cp_Cap05141	11.674	-	-	Phosphatase iiic
Cp_Cap00078	11.573	-	4.445	Acid phosphatase
Cp_Cap13164	11.387	-	-	-
Cp_Cap04682	-	7.907	11.350	Peroxidase
Cp_Cap03000	11.342	-	-	Hypothetical protein
Cp_Cap07565	-	10.738	9.002	oxidoreductase
Cp_Cap05172	-	9.733	10.614	NAD binding Rossmann fold oxidoreductase, putative
Cp_Cap13216	-	10.406	8.793	subtilase
Cp_Cap04214	-	8.306	10.320	hydrolase activity
Cp_Cap11815	-	10.172	10.237	hypothetical protein
Cp_Cap07299	9.919	8.728	5.375	hydrolase activity
Cp_Cap12528	9.518	7.554	5.035	hypothetical protein
Cp_Cap04450	-	5.319	9.472	3-oxoacyl-[acyl-carrier protein] reductase
Cp_Cap05866	-	-	9.411	hypothetical protein
Cp_Cap14295	-	5.582	9.397	endopolygalacturonase

**Fig 3:**
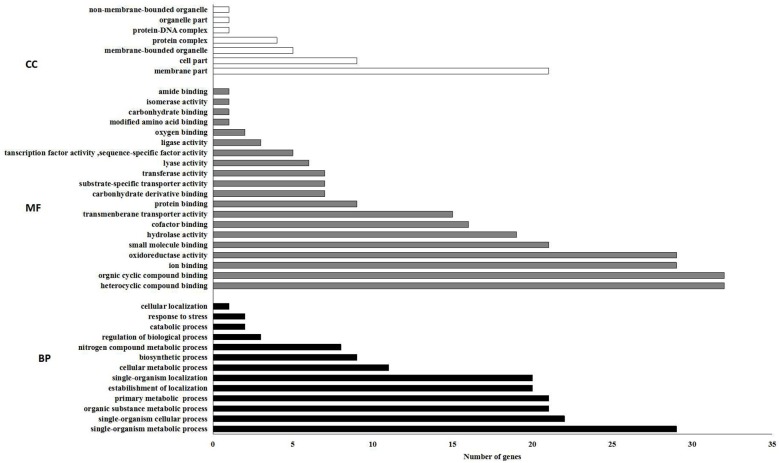
The 20 most enriched GO terms. Bar chart of DEGs enriched in GO term; it can directly reflect the number of DEGs distributing into different GO terms. CC, cellular component; MF, molecular function; BP, biological process.

GO enrichment analysis of DEGs showed that oxidoreductase, hydrolase, and transmembrane transporter activities were significantly enriched in the molecular function category (Fig 3). The membrane part was the dominant subcategory of the cellular component category.

### qRT-PCR verification

**Fig 4:**
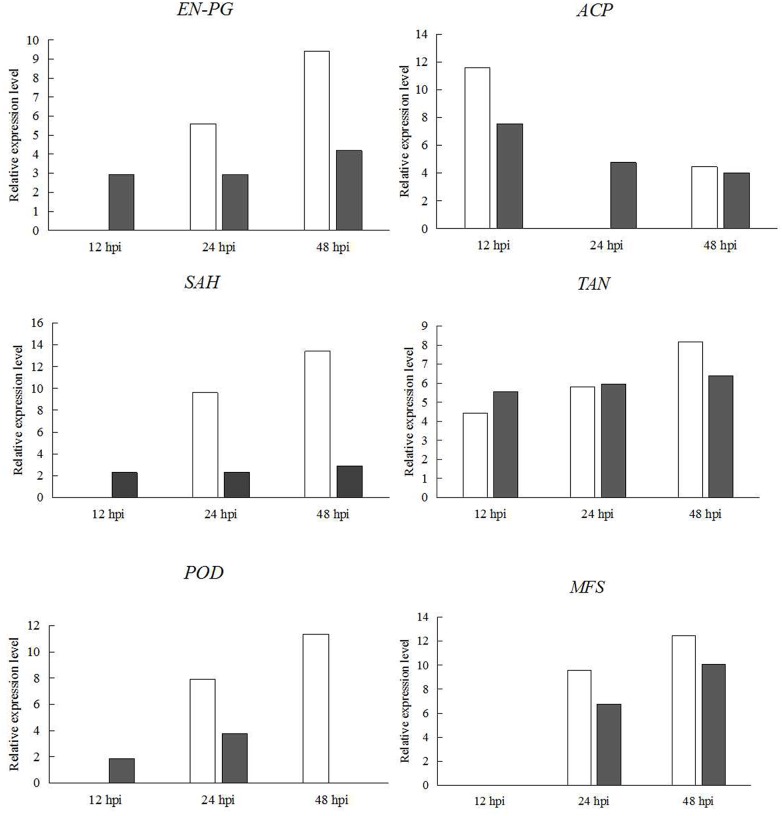
Validation of DEGs by qRT-PCR analysis. The relative expression levels of six differential expressed genes in *C*. *pseudoreteaudii* at 12 hpi, 24 hpi and 48 hpi of *Eucalyptus* leaves were obtained by RNA-seq (white) and by qRT-PCR (black). *EN-PG*, endopolygalacturonase; *ACP*, acid phosphatase; *POD*, peroxidase; *SAH*, salicylate hydroxylase; *TAN*, tannase; *MFS*, major facilitator superfamily transporter; *BGC*, beta-glucosidase G. The y-axis standed for the relative expression levels [log2(foldchange)]. Error bars indicated standard error of mean of three biological replicates.

qRT-PCR analysis showed that the expression changes of all genes were consistent with those showed by RNA-seq (Fig 4), despite some differences in the degree of the changes. It confirmed that the RNA-seq results were valid.

## Discussion

*Eucalyptus* leaf blight caused by *Calonectria* spp is one of the most severe diseases in the *Eucalyptus* plantation and nursery. In this study, we investigated the expression profiles of *C*. *pseudoreteaudii* during infection with *Eucalyptus* and cultured on PDB medium by RNA-seq. We identified 161 DEGs of *C*. *pseudoreteaudii* at three stages of infection (S2 File). These DEGs are involved in cell wall degradation, detoxification of phytoalexins, toxin synthesis, iron uptake, ROS scavenging, and so on.

### Cell wall degrading enzymes

The epidermis and cell wall are the main physical barriers for plants that prevent pathogen invasion. They are composed of cutin, cellulose, hemicellulose, pectin, lignin, and structural proteins. Phytopathogens secrete a series of cell wall degrading enzymes (CWDEs) to facilitate host cell wall degradation and promote infection [28]. These enzymes include cutinase, cellulase, hemicellulase, pectinase, aprotease, and so on. They are required for all the phytopathogens to degrade plant tissues and get nutrition at the later stage of invasion. Moreover, they are particularly important for pathogens that directly penetrate the host epidermis.

Our study showed that several CWDE genes of *C*. *pseudoreteaudii* were differentially expressed during *Eucalyptus* leaf infection. For example, one cutinase gene (Cp_Cap05169) was upregulated at the early stage. Cutinase is a crucial pathogenic factor for surface-penetrating phytopathogenic fungi such as *Magnaporthe grisea* [29]. Deletion of cutinase gene will reduce the pathogenicity of *Curvularia lunata* on unwounded maize (*Zea mays*) leaves [30]. The extracellular cutinase Pbc1 is also required for pathogenicity of *Pyrenopeziza brassicae* on rape seed [31]. Genes encoding cutinase are also expressed earlier than other CWDEs because they are involved in the hydrolysis of the cuticle, which is the outermost protection structure of plant [32]. Moreover, our preliminary studies showed that the thickness of the cuticle was one of the important indexes of *Eucalyptus* against *Calonectria* [19]. The high expression of cutinase gene at the early infection stage in this study suggested that it was an important pathogenic factor for *Calonectria* to facilitate the infection of *Eucalyptus*. Besides, one endopolygalacturonase (endo-PG) gene (Cp_Cap14295) was significantly upregulated at the middle and later infection stage. Endo-PG was secreted by the phytopathogen to hydrolyze pectin and macerate plant tissue. The deletion of single one endo-PG gene could reduce virulence in tomato. Although the single deletion mutants of PG genes in *Fusarium oxysporum* did not display a significant virulence reduction on tomato plants, the virulence of *Δpg1Δpgx6* double mutant was significantly attenuated [33]. Several genes involved in the degradation of cellulose and hemicellulose such as beta-galactosidase (Cp_Cap06841), beta-glucosidase (Cp_Cap12678) were also observed to continuously upregulate during infection on *Eucalyptus* leaves.

In plant cell walls, ferulic acid (FA) residues are linked mainly to xylan and pectin, which cross-link cell wall polymers like lignin and cellulose. This increases the physical strength and integrity of plant cell walls and decreases their biodegradability by microorganisms. The linkage between two FA molecules on adjacent polysaccharide chains is the most important cross-linkage in plant cell walls. Feruloyl esterases act synergistically with other esterases to remove these linkages and facilitate the breakdown of complex plant cell wall carbohydrates [34]. We detected the continuous high expression level of feruloyl esterase gene (Cp_Cap00122) in *C*. *pseudoreteaudii* during infection. However, two feruloyl esterase genes, *FAEB1* and *FAED1*, were not necessary for the pathogenicity of *F*. *graminearum* on wheat [35]. It would be interesting to explore the role of feruloyl esterase in the virulence of *C*. *pseudoreteaudii*.

### Detoxification of phytoalexins

*Eucalyptus* species employ an array of secondary metabolites as phytoalexins against biotic stresses including terpenoids and phenolic compounds. In our previous study, the content of polyphenols and flavonoids in *Eucalyptus* increased after *C*. *pseudoreteaudii* infection and increased more in resistant cultivars than in susceptible ones [16]. However, for the pathogen that successfully invade and colonize the host, the detoxification processes of phytoalexins in pathogens are far from understood.

One of the detoxification approaches is to metabolize the phytoalexin by hydration, oxidation, oxidative dimerization, and glycosylation. For example, CfTom1, a glycosyl hydrolase (GH10) orthologous to fungal tomatinases, is responsible for the detoxification of α-tomatine by *Cladosporium fulvum* and is required for full virulence of this fungus on tomato [36]. Transporters in phytopathogenic fungi also play a significant role in detoxifying the phytoalexin. It can weaken or neutralize the effects of phytoalexins by extruding toxic compounds out of the cell or segregating them into vacuole [37]. Our results showed that one hydrolase gene (Cp_Cap14347) and one transporter gene (Cp_Cap12745) relating with the detoxification of phytoalexin were upregulated during the *Calonectria* infection in *Eucalyptus*. They may give the pathogen the ability to detoxify the phytoalexin of *Eucalyptus*.

### Candidate effectors

A large number of pathogenic effectors share features as secreted and small cysteine-rich proteins (SSPs) [38]. The disulfide bond of partial cysteine residues maintains the structure and function of these proteins when transferred into host cells. These proteins play important roles in mediating pathogen-host interactions. For example, a small cysteine-rich protein SsSSVP1 in the necrotrophic phytopathogen *Sclerotinia sclerotiorum* may manipulate plant energy metabolism to facilitate the infection of *S*. *sclerotiorum* by interaction with QCR8, a subunit of the cytochrome b-c1 complex of mitochondrial respiratory chain in plants [39]. Another small cysteine-rich secretory protein, SCR96, from *Phytophthora cactorum*, was transcriptionally induced throughout the developmental and infection stages [40]. It can trigger cell death in the Solanaceae and is important for pathogenicity and oxidative stress tolerance. We found that five SSPs whose lengths were shorter than 300 amino acids and had at least four cysteine residues in the mature proteins were differentially expressed during infection of *Eucalyptus*. Three of them were significantly upregulated at the early stage. These proteins may participate in the interaction between *Calonectria* and *Eucalyptus*.

### Proteins involved in the iron uptake

Fungal pathogens have evolved at least two pathways for iron uptake from its host's iron-limiting environment, siderophore-mediated iron uptake and reductive iron assimilation [41]. Siderophore-mediated iron uptake is the major pathway of cellular iron uptake for some fungi [42]. This requires synthesis and excretion of siderophore, which is a low-molecular weight (Mr < 1500) and high-affinity ferric iron-specific chelator. Siderophore is required for full virulence in many plant-pathogens interactions [42,43]. L-ornithine N^5^-oxygenase is the first enzyme that catalyses the N-hydroxylation of l-ornithine in the synthesis pathway of siderophore. Nonribosomal peptide synthetases (NRPSs) link the hydroxamate groups and sometimes other amino acids to complete siderophore biosynthesis. Mutants of these two genes are short of siderophore, and decrease the ability to cope with oxidative stress and the virulence on hosts [44]. We found that three genes related to iron absorption were upregulated during infection on *Eucalyptus* leaves. Among these, one gene (Cp_Cap02183) encoding L-ornithine N^5^-oxygenase is homologous to the siderophore biosynthetic gene *SID1* which is required for full virulence of *F*. *graminearum* on wheat [42]. It was suggested that enhancing transportability of iron may facilitate *C*. *pseudoreteaudii* infection of *Eucalyptus*.

### Enzymes involved in toxin synthesis

Polyketides are a diverse group of secondary metabolites produced by microorganisms and some of them like mycotoxins, are crucial for necrotrophic and hemibiotrophic pathogens to establish a compatible interaction during their necrotrophic stage [45]. Polyketides are synthesized by complex enzymatic systems called polyketide synthases (PKS) and modifying enzymes such as cytochrome P450s [46]. The present data showed that three DEGs (versicolorin B synthase, putative conidial pigment polyketide synthase PksP/Alb1, putative cytochrome p450 protein) about polyketides synthesis were found in *C*. *pseudoreteaudii* during the infection. Versicolorin B synthase is a key enzyme involved in the synthesis of versicolorin produced by *Aspergillus flavus* and *A*. *parasiticus* [47].

### Scavenger of ROS

Reactive oxygen species (ROS) are produced by plant cells during pathogen infections. It protects plants from pathogen attack by eliciting a hypersensitive response or doing direct harm to the pathogen, or inducing the production of phytoalexin, and so on. To counteract ROS, pathogens are equipped with an antioxidant system. This antioxidant system includes enzymes such as catalases, peroxidases (POD), and superoxide dismutases (SODs) [48]. Three POD genes (Cp_Cap04682, Cp_Cap03142, Cp_Cap09760) of *C*. *pseudoreteaudii* were upregulated at different stages of infection. Perhaps, they are secreted by *C*. *pseudoreteaudii* to scavenge the ROS produced by *Eucalyptus* to favor infection.

### Other virulence factors

Phosphatase is an enzyme dephosphorylating signaling protein that plays a key role not only in physiological cell functions of an organism but also in host-pathogen interactions [49]. Pathogens secrete phosphatases into the host cell for changing the phosphorylation level of the host cell. This facilitates the invasion and colonization of the pathogen by adjusting the metabolic system of the host cell or interfering with the defense response of the host [50]. For the pathogen who is unable to absorb the organic phosphorus compounds from the host plant, phosphatase can break down these compounds into small molecules that can be directly utilized by the pathogen. The upregulation of two phosphatase genes (Cp_Cap00078, Cp_Cap05141) of *C*. *pseudoreteaudii* indicated their potential role in the interaction between *C*. *pseudoreteaudii* and *Eucalyptus*.

### Conclusions

**Fig 5:**
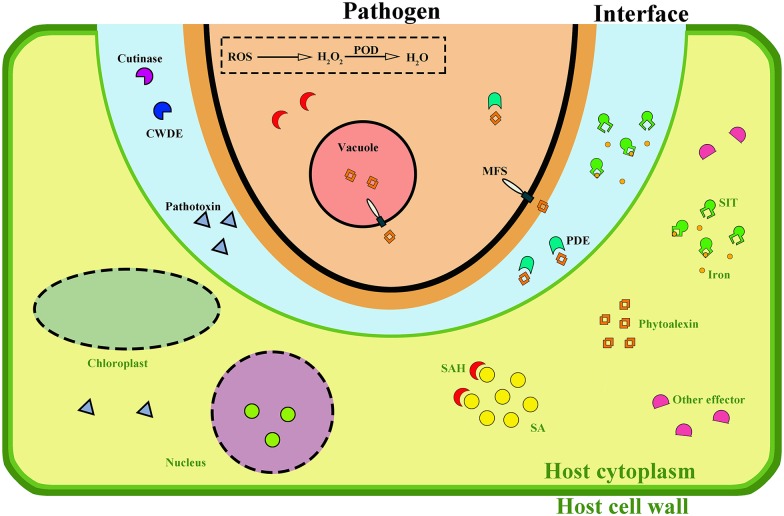
Mode of hypha action of *C*. *pseudoreteaudii* on host cell. The fungal plasma membrane is shown in black, the fungal cytoplasm is shown in pink, the plant plasma membrane is shown in light green, and the plant cell wall is shown in dark green. The interface between the fungal hypha and the plant plasma membrane is shown as a light blue area. Fungal effectors and targeted plant substrate are shown in various colors. CWDE, cell wall degrading enzyme; PDE, phytoalexin degrading enzyme; ROS, reactive oxygen species; POD, peroxidase; MFS, major facilitator superfamily; SIT, siderophore iron transporter; SAH, salicylate hydroxylase; SA, salicylic acid.

In this paper, comparative transcriptome analysis of *C*. *pseudoreteaudii* during infection on *Eucalyptus* were carried out by RNA-seq. This paper is the first attempt to demonstrate the molecular and pathogenic mechanism of *Calonectria* at different infection stage. The results showed that genes involved in cell wall degradation, detoxification of phytoalexins, toxin synthesis were significantly upregulated. Further changes were found in iron uptake and ROS scavenging. In a word, we speculated that *C*. *pseudoreteaudii* may secrete a series of cell wall degrading enzymes to facilitate infection at different stages (Fig 5). For example, it initially produced cutinases to break through the cuticle of the plant epidermis, and also pectinase, cellulase and hemicellulase to degrade host complex carbon sources for infection and nourishment. Toxic metabolites may be another crucial weapon for this pathogen to induce host cell death and promote infection. After invading into the host cell, this pathogen suffered stress under the toxicity of *Eucalytpus* phytoalexin, but it could survive under the host hostile environment by an effective detoxification system including rapidly degradation metabolism and sequestration to the vacuoles. In addition, several candidate genes were observed to upregulated at the early stages. Further studies were required to determine the function of these genes. These findings provided insight into the pathogenic mechanisms of *Calonectria* and the development of a better control of this disease.

## Supporting Information

S1 File qRT-PCR primer sequences for six DEGs. (XLS)

S2 File Differentially expressed genes in *C*. *pseudoreteaudii* during infection of *Eucalyptus*. (XLS)
